# Supplementary figures and images for: Landscape composition and local floral resources influence foraging behavior but not the size of Bombus impatiens Cresson (Hymenoptera: Apidae) workers
Source: PLoS One. 2020 Jun 25;15(6):e0234498. doi: 10.1371/journal.pone.0234498 (PMC7316238; doi:10.1371/journal.pone.0234498)

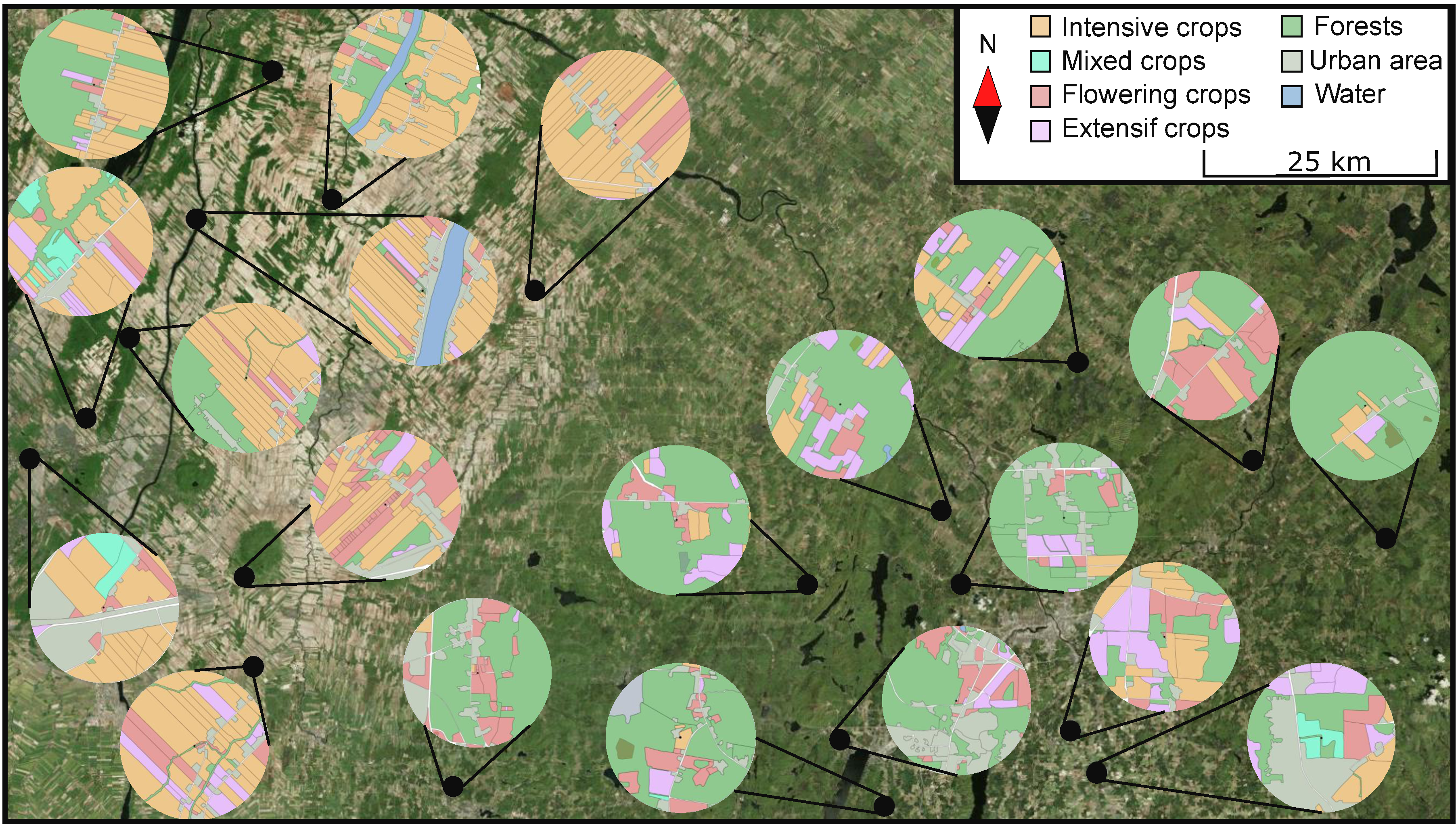

Supplement: S1 Fig — (TIF) [file pone.0234498.s001.tif]

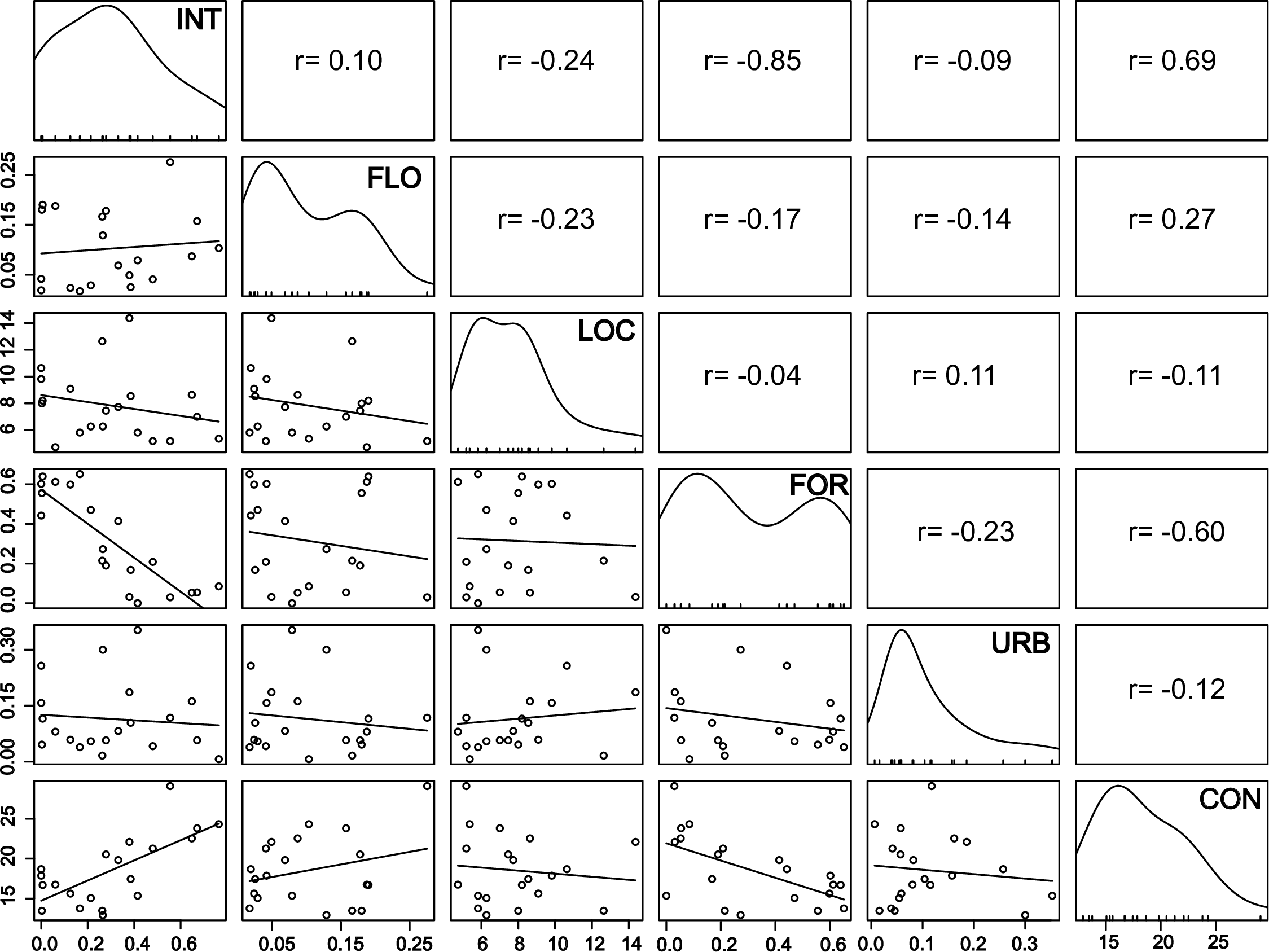

Supplement: S2 Fig — (TIF) [file pone.0234498.s002.tif]
