# Supplementary material for: Landscape composition and local floral resources influence foraging behavior but not the size of Bombus impatiens Cresson (Hymenoptera: Apidae) workers
Source: PLoS One. 2020 Jun 25;15(6):e0234498. doi: 10.1371/journal.pone.0234498 (PMC7316238; doi:10.1371/journal.pone.0234498)
Supplement: S1 Table — (DOCX) [file pone.0234498.s003.docx]

Table S1. ∆AICc comparing both scale (500m vs 1000m) for all models.

| Models | ∆AICc 500m | ∆AICc 1000m |
| --- | --- | --- |
| Weight | 0 | 0.84 |
| Thorax | 2.07 | 0 |
| Wing | 0 | 0.92 |
| Head length | 0 | 0.98 |
| Tibia | 0 | 2.11 |
| Food-provisioning Activty | 0 | 0.10 |
| Pollen load | 0 | 0.46 |
| Distance | 0 | 11.31 |
| **Mean** | **0.26** | **2.09** |
