# Supplementary material for: Landscape composition and local floral resources influence foraging behavior but not the size of Bombus impatiens Cresson (Hymenoptera: Apidae) workers
Source: PLoS One. 2020 Jun 25;15(6):e0234498. doi: 10.1371/journal.pone.0234498 (PMC7316238; doi:10.1371/journal.pone.0234498)
Supplement: S2 Table — # workers represent the number of workers carrying this pollen (n = 264) and the average relative abundance, in percentage. (DOCX) [file pone.0234498.s004.docx]

Table S2. List of the plants found in the pollen loads of workers. # workers represent the number of workers carrying this pollen (n= 264) and the average relative abundance, in %.

| Family | Taxa | # workers | Relative abundance (%) |
| --- | --- | --- | --- |
| Pinophyta | Pinophyta | 4 | 0.02 |
| Adoxaceae | *Sambucus* Linnaeus | 17 | 0.19 |
|  | *Sambucus canadensis* Linnaeus | 7 | 1.90 |
|  | *Sambucus racemosa* Linnaeus | 1 | 0.17 |
|  | *Viburnum* Linnaeus | 19 | 6.40 |
| Amaranthaceae | *Chenopodium* Linnaeus | 9 | 0.99 |
|  | *Chenopodium album* Linnaeus | 1 | 0.03 |
| Anacardiaceae | *Rhus typhina* Linnaeus | 18 | 12.61 |
| Apiaceae | *Anthriscus sylvestris* (Linnaeus) Hoffmann | 18 | 6.15 |
| Aquifoliaceae | *Ilex mucronata* (Linnaeus) M. Powell, V. Savolainen & S. Andrews | 2 | 4.49 |
| Asparagaceae | *Maianthemum*F.H. Wiggers | 1 | 100 |
| Asteraceae | *Ambrosia artemisiifolia* Linnaeus | 3 | 4.67 |
|  | Asteraceae Berchtold & J. Presl | 1 | 0.04 |
|  | Type- *Taraxacum officinale* | 5 | 0.08 |
|  | *Centaurea nigra* Linnaeus | 2 | 7.1 |
|  | *Erigeron*Linnaeus | 18 | 8.33 |
|  | *Hieracium* Linnaeus | 2 | 50 |
|  | *Leucanthemum vulgare* Lamarck | 12 | 1.32 |
|  | *Sonchus* Linnaeus | 13 | 1.87 |
|  | *Taraxacum officinale* F.H. Wiggers | 124 | 18.43 |
|  | *Solidago* Linnaeus | 1 | 0.61 |
| Betulaceae | *Betula*Linnaeus | 18 | 3.78 |
| Boraginaceae | *Lappula squarrosa* (Retzius) Dumortier | 1 | 50 |
|  | *Myosotis* Linnaeus | 1 | 0.57 |
| Brassicaceae | Brassicaceae Burnett | 39 | 2.67 |
| Caprifoliaceae | *Lonicera tatarica* Linnaeus | 22 | 17.41 |
|  | *Valeriana officinalis* Linnaeus | 2 | 52.13 |
| Caryophyllaceae | *Silene flos-cuculi* (Linnaeus) Clairville | 16 | 14.58 |
| Convolvulaceae | *Calystegia sepium* (Linnaeus) R. Brown | 1 | 4.8 |
| Cornaceae | *Cornus* Linnaeus | 2 | 0.05 |
|  | *Cornus sericea* Linnaeus | 36 | 8.99 |
| Cyperaceae | *Carex* Linnaeus | 2 | 0.35 |
| Ericaceae | Ericaceae de Jussieu | 22 | 2.66 |
| Fabaceae | *Lotus corniculatus* Linnaeus | 3 | 6.21 |
|  | *Melilotus* Miller | 20 | 9.57 |
|  | *Trifolium campestre* Schreber | 1 | 0.45 |
|  | *Trifolium hybridum* Linnaeus | 13 | 8.82 |
|  | *Trifolium pratense* Linnaeus | 9 | 15.74 |
|  | *Trifolium repens* Linnaeus | 37 | 9.46 |
|  | *Trifolium* Linnaeus | 1 | 7.55 |
|  | *Medicago sativa* Linnaeus | 1 | 81.76 |
|  | *Vicia cracca*Linnaeus | 72 | 22.71 |
| Hypericaceae | *Hypericum* Linnaeus | 3 | 0.61 |
| Lamiaceae | Lamiaceae Martinov | 1 | 0.004 |
|  | Type- *Mentha* Linnaeus | 14 | 30.55 |
| Liliaceae | Liliaceae de Jussieu | 7 | 22.27 |
|  | *Lilium canadense* Linnaeus | 1 | 0.67 |
| Lythraceae | *Lythrum salicaria* Linnaeus | 1 | 0.65 |
| Malvaceae | *Tilia* Linnaeus | 11 | 19.24 |
| Myricaceae | Type*-Comptonia peregrina* (Linnaeus) J.M. Coulter | 1 | 11.11 |
| Oleaceae | *Fraxinus* Linnaeus | 19 | 3.79 |
| Oxalidaceae | *Oxalis* Linnaeus | 16 | 22.88 |
| Pinaceae | *Picea* A. Dietrich | 116 | 5.30 |
| Plantaginaceae | *Plantago* Linnaeus | 22 | 10.05 |
|  | *Veronica* Linnaeus | 2 | 15.81 |
| Poaceae | Poaceae Barnhart | 69 | 3.28 |
| Polygonaceae | *Rumex*Linnaeus | 4 | 0.02 |
| Primulaceae | *Lysimachia borealis* (Rafinesque) U. Manns & Anderberg | 1 | 53.91 |
| Ranunculaceae | *Ranunculus acris*Linnaeus | 28 | 4.26 |
|  | *Thalictrum pubescens* Pursh | 4 | 19.05 |
| Rhamnaceae | *Rhamnus cathartica* Linnaeus | 9 | 11.29 |
|  | *Frangula alnus* Miller | 14 | 6.13 |
|  | *Rhamnus*Linnaeus | 1 | 11.39 |
| Rosaceae | *Amelanchier* Medikus | 1 | 1.33 |
|  | *Crataegus* Linnaeus | 4 | 0.34 |
|  | *Fragaria* Linnaeus | 20 | 28.43 |
|  | *Geum* Linnaeus | 30 | 17.66 |
|  | *Malus* Miller | 53 | 0.83 |
|  | *Potentilla norvegica* Linnaeus | 2 | 18 |
|  | *Potentilla* Linnaeus | 5 | 0.77 |
|  | *Prunus pensylvanica* Linnaeus f. | 17 | 8.70 |
|  | *Prunus* Linnaeus | 5 | 0.89 |
|  | *Prunus virginiana* Linnaeus | 6 | 3.76 |
|  | *Rubus allegheniensis* Porter | 41 | 29.75 |
|  | *Rubus idaeus* Linnaeus | 2 | 42.26 |
|  | *Rubus pubescens* Rafinesque | 3 | 83.11 |
|  | *Rubus* Linnaeus | 68 | 27.53 |
|  | *Spiraea*Linnaeus | 13 | 13.26 |
| Rubiaceae | *Galium* Linnaeus | 5 | 1.67 |
| Salicaceae | *Populus* Linnaeus | 1 | 1.25 |
|  | *Salix* Linnaeus | 129 | 26.43 |
| Sapindaceae | *Acer negundo* Linnaeus | 1 | 2.79 |
|  | *Acer platanoides* Linnaeus | 3 | 25.79 |
|  | *Acer rubrum* Linnaeus | 4 | 34.36 |
|  | *Acer saccharinum* Linnaeus | 1 | 100 |
|  | *Acer* Linnaeus | 13 | 32.93 |
|  | *Acer spicatum* Lamarck | 8 | 4.05 |
|  | *Aesculus hippocastanum* Linnaeus | 2 | 4.71 |
| Scrophulariaceae | *Verbascum thapsus* Linnaeus | 2 | 13.37 |
| Solanaceae | *Solanum dulcamara* Linnaeus | 39 | 22.51 |
| Typhaceae | *Typha* Linnaeus | 1 | 0.60 |
| Vitaceae | *Vitis* Linnaeus | 60 | 25.91 |
|  | *Parthenocissus* Planchon | 1 | 2.67 |
|  | Unknown 1 | 1 | 4.08 |
|  | Unknown 2 | 1 | 98.68 |
|  | Unknown 3 | 1 | 97.33 |
|  | Unknown 4 | 1 | 16.88 |
|  | Unknown 5 | 4 | 24.80 |
|  | Unknown 6 | 1 | 2.99 |
|  | Unknown 7 | 1 | 22.16 |
|  | Unknown 8 | 2 | 8.99 |
|  | Unknown 9 | 1 | 21.75 |
|  | Unknown 10 | 6 | 76.05 |
|  | Unknown 11 | 1 | 5.56 |
|  | Unknown 12 | 1 | 12.33 |
|  | Unknown 13 | 1 | 1.27 |
|  | Unknown 14 | 1 | 0.03 |
|  | Unknown 15 | 1 | 0.26 |
|  | Unknown 16 | 2 | 0.29 |
|  | Unknown 17 | 1 | 0.18 |
|  | Unknown 18 | 1 | 0.02 |
|  | Unknown 19 | 2 | 0.17 |
|  | Unknown 20 | 2 | 0.16 |
|  | Unknown 21 | 1 | 0.01 |
|  | Unknown 22 | 1 | 0.06 |
|  | Unknown 23 | 1 | 0.13 |
|  | Unknown 24 | 1 | 0.20 |
|  | Unknown 25 | 1 | 0.04 |
|  | Unknown 26 | 1 | 0.07 |
|  | Unknown 27 | 2 | 0.21 |
|  | Unknown 28 | 1 | 0.02 |
|  | Unknown 29 | 1 | 0.004 |
|  | Unknown 30 | 3 | 6.69 |
|  | Unknown 31 | 1 | 1.25 |
|  | Unknown 32 | 2 | 3.08 |
|  | Unknown 33 | 1 | 22.78 |
|  | Unknown 34 | 2 | 48.43 |
|  | Unknown 35 | 1 | 0.62 |
