# Supplementary material for: Landscape composition and local floral resources influence foraging behavior but not the size of Bombus impatiens Cresson (Hymenoptera: Apidae) workers
Source: PLoS One. 2020 Jun 25;15(6):e0234498. doi: 10.1371/journal.pone.0234498 (PMC7316238; doi:10.1371/journal.pone.0234498)
Supplement: Attachment — Submitted filename: PONE-D-19-31905_R1_Effects of local and landscape factors on body size and foraging in B.impatiens.docx [file pone.0234498.s005.docx]

Reviewer: Heather Grab

First, I hope that both the journal staff, authors and their families are safe as we all face the challenges of covid-19.

The manuscript presents results of a large field study which evaluates the impacts of local and landscape-scale resources on individual forager traits and on colony-level foraging activity. I was very excited to see this study as my own research is at the intersection of intraspecific morphological variation and landscape ecology. The authors have done a good job addressing previous reviewer comments; however, I have some additional points that the authors should address before publication.

Major comments:

I found it very difficult to interpret the results from the model comparisons reported by the authors. As I understand, the authors conducted comparisons based on a pre-defined set of candidate models. They explained why some landcovers (border, urban, forest) were excluded from their models, however others (extensive covers) seem to be left out without justification even though they comprise a substantial portion (~50%) of the landscape at some sites. Table 2 reports Model-averaged coefficients but which model sets were used to obtain these averages is unclear as they appear to be reported on an individual model basis? In table three, I suggest labeling each candidate model by its predictors as in Table 1, because it is currently very difficult to understand the differences between models.

Given these difficulties, I was very happy to see that the author’s provided a Dryad link to the underlying data supporting their analysis. However, when looking at the data I noticed a few issues that the authors can hopefully resolve:

First, the authors report in their methods that foraging bees were only collected starting four weeks after the colonies were set out at each site (May 3). This makes a lot of sense, as the goal of the study was to measure variation in workers relative the resources available at a sites, and therefore the authors would want to be sure that they were measuring workers actually provisioned at the site rather than those that came with the colony or were provisioned primarily with the nectar and pollen resources provided to the colony during factory rearing. However, in looking at the worker datafile, many workers appear to be collected before this 4 week period (May 18-31^st^). After excluding these data, I no longer see a significant effect of JJ (Julian date) on the morphological measures.

> R Output
Read in data and add new variable that describe total agricultural cover consistent with landscape analyses of other recent studies

setwd("~/Downloads")

bee=read.delim("workers.csv", sep=",")

lc=read.delim("landscape_variables.csv", sep=",")

lc$AG_500=lc$FLO_500+lc$INT_500 *#high and low pesticide cultivated land @ 500m*

lc$AG_1000=lc$FLO_1000+lc$INT_1000 *#high and low pesticide cultivated land @ 1000m*

Merge the datasets and create a subset excluding individuals collected before the 4 week window Additionally, add a new column to assess the number of samples per site

```
dat=bee %>%
  full_join(lc) %>%
  group_by(Site) %>%
  add_tally() %>%
  select(Site,Hive,n,everything())
## Joining, by = "Site"
dat_sub=bee %>%
  full_join(lc) %>%
  group_by(Site) %>%
  filter(date>160531) %>%
  add_tally() %>%
  select(Site,Hive,n,everything())
## Joining, by = "Site"
```

Evaluate models on morphological traits

First, how does sample start date influence body size over time trend

m=lmer(thorax_avg~scale(JJ)+(1|Site), data=dat)

anova(m)

|  | **Sum Sq**  <dbl> | **Mean Sq**  <dbl> | **NumDF**  <int> | **DenDF**  <dbl> | **F value**  <dbl> | **Pr(>F)**  <dbl> |
| --- | --- | --- | --- | --- | --- | --- |
| scale(JJ) | 0.5091212 | 0.5091212 | 1 | 202.9498 | 5.428469 | 0.02079389 |

1 row

m=lmer(thorax_avg~scale(JJ)+(1|Site), data=dat_sub)

anova(m)

|  | **Sum Sq**  <dbl> | **Mean Sq**  <dbl> | **NumDF**  <int> | **DenDF**  <dbl> | **F value**  <dbl> | **Pr(>F)**  <dbl> |
| --- | --- | --- | --- | --- | --- | --- |
| scale(JJ) | 0.006727585 | 0.006727585 | 1 | 139.8781 | 0.06970135 | 0.7921607 |

1 row

The effect of date is no longer significant after accounting for the 4 week window. This suggest that the effect was largely due to workers produced before the start of the study and that worker size is smaller once colonies are placed in the field.

In running some additional models with the Extensive cover variable on the reduced dataset, I found that an interaction with local flower cover did explain significant variation in several of the morphological traits (thorax shown here as an example). Using the lme4 and lmerTest packages I was able to account for the nested random effect of Hive within Site without running into model singularity or convergence issues. As the majority of variables considered by the author are continuous and have this hierarchical structure, I recommend using this package rather the glmmTMB. Additionally, the data provided by the authors appears to only have the morphological measures for the bees which had weight data taken (205 rather than the full 264 reported in the results). Because this is actually quite a small sample size given the date range, number of colonies and sites sampled, I evaluated whether there were sites with only a few bees collected (<5) and removed them from the analysis. This ensures that results describe the patterns only for sites with a sample sufficient to accurately characterize the average size of workers within a site.

m=lmer(thorax_avg~EXT_500*LOC_10w+(1|Site/Hive), data=subset(dat_sub, n>=5))

anova(m)

|  | **Sum Sq**  <dbl> | **Mean Sq**  <dbl> | **NumDF**  <int> | **DenDF**  <dbl> | **F value**  <dbl> | **Pr(>F)**  <dbl> |
| --- | --- | --- | --- | --- | --- | --- |
| EXT_500 | 0.6074131 | 0.6074131 | 1 | 13.24730 | 6.241334 | 0.02638081 |
| LOC_10w | 0.1179987 | 0.1179987 | 1 | 18.41288 | 1.212469 | 0.28503049 |
| EXT_500:LOC_10w | 0.4719130 | 0.4719130 | 1 | 13.12365 | 4.849033 | 0.04613809 |

3 rows

plot_model(m, type="int", show.data = F, mdrt.values = "meansd")

These results suggest that covers like pasture and hay fields have a negative effect on body size when local flower resources are low but that high floral resources buffer against this negative effect. These results would be consistent with Grab et al 2019 JAE.

Additionally, I assessed whether the aggregate cover of both flowering and intensive crops had an impact on morphological measures

m=lmer(worker_weight~AG_1000*LOC_10w+(1|Site/Hive), data=subset(dat_sub, n>=5))

anova(m)

|  | **Sum Sq**  <dbl> | **Mean Sq**  <dbl> | **NumDF**  <int> | **DenDF**  <dbl> | **F value**  <dbl> | **Pr(>F)**  <dbl> |
| --- | --- | --- | --- | --- | --- | --- |
| AG_1000 | 0.0011134459 | 0.0011134459 | 1 | 16.04991 | 4.377851 | 0.05265427 |
| LOC_10w | 0.0009163111 | 0.0009163111 | 1 | 15.29444 | 3.602755 | 0.07672137 |
| AG_1000:LOC_10w | 0.0011518682 | 0.0011518682 | 1 | 15.94197 | 4.528920 | 0.04927795 |

3 rows

plot_model(m, type="int", show.data = F, mdrt.values = "meansd")

These results suggest that then local floral resources are high, worker weight declines with greater ag cover but that ag cover can have the opposite effect, increasing body size when local floral resources are low.

I am also concerned with the use of a correlation test when evaluating whether body size influences the number of pollen types carried by foragers. First because foragers are not all independent (multiple foragers from the same sites) but more importantly because local floral resources vary across sites, and if above analyses hold true, then variation in floral resources is related to body size in at least some landscape conditions. Because pollen richness is count data, I recommend a poisson glm model.

Proposed model structure for pollen richness vs body size analysis. I used bees from all dates and did not set a minimum replication threshold.

```
m=glmer(Pollen_load_richness~LOC_10w+scale(thorax_avg)+(1|Site/Hive), data=subset(dat, ), family="poisson")
summary(m)
## Generalized linear mixed model fit by maximum likelihood (Laplace
##   Approximation) [glmerMod]
##  Family: poisson  ( log )
## Formula: Pollen_load_richness ~ LOC_10w + scale(thorax_avg) + (1 | Site/Hive)
##    Data: subset(dat, )
##
##      AIC      BIC   logLik deviance df.resid
##   1170.1   1186.7   -580.0   1160.1      200
##
## Scaled residuals:
##     Min      1Q  Median      3Q     Max
## -2.4051 -0.9661 -0.2117  0.7685  5.4298
##
## Random effects:
##  Groups    Name        Variance Std.Dev.
##  Hive:Site (Intercept) 0.10302  0.3210
##  Site      (Intercept) 0.01575  0.1255
## Number of obs: 205, groups:  Hive:Site, 76; Site, 20
##
## Fixed effects:
##                   Estimate Std. Error z value Pr(>|z|)
## (Intercept)        1.45164    0.18518   7.839 4.54e-15 ***
## LOC_10w            0.03019    0.02202   1.371  0.17037
## scale(thorax_avg) -0.10057    0.03242  -3.102  0.00192 **
## ---
## Signif. codes:  0 '***' 0.001 '**' 0.01 '*' 0.05 '.' 0.1 ' ' 1
##
## Correlation of Fixed Effects:
##             (Intr) LOC_10
## LOC_10w     -0.952
## scl(thrx_v)  0.012  0.004
```

Results indicate that larger individuals still collect fewer pollen types even after accounting for repeated measures within sites and for site level variation in floral richness

The authors should certainly re-run each of these models with their full dataset. Because the authors did not provide the foraging activity data, I would encourage them to reassess these models as well.

L311-312: while the authors are correct that pollen load richness declines increasing proportions of intensive crop cover when local floral richness is low it does not follow from the data to suggest that when local floral richness is high, pollen load richness increases with intensive crop cover. The pattern in figure 3 suggests that this relationship is likely not different than zero.

Minor comments:

L67-83 discussion of how differences in metric limits synthesis seems outside of the scope of the current work given that the authors are not attempting a synthesis effort. I would limit this section only to explaining why particular metrics might be expects to impact body size and foraging variation.

L130-131: It would be very helpful if the authors would provide a map of their study sites, based on their regional description it seems that spatial autocorrelation many be an issue. Given this concern, I suggest that the authors confirm that model residuals do not display spatial autocorrelation.
